# Participatory and Spatially Explicit Assessment to Envision the Future of Land-Use/Land-Cover Change Scenarios on Selected Ecosystem Services in Southwestern Ghana

**DOI:** 10.1007/s00267-024-01943-z

**Published:** 2024-02-28

**Authors:** Evelyn Asante-Yeboah, HongMi Koo, Mirjam A. F. Ros-Tonen, Stefan Sieber, Christine Fürst

**Affiliations:** 1 Department for Sustainable Landscape Development, Martin-Luther-University, Halle-Wittenberg, Germany; 2 Leibniz Centre for Agricultural Landscape Research (ZALF), 15374, Müncheberg, Germany; 3 German Centre for Integrative Biodiversity Research (iDiv), Halle-Jena-Leipzig, Leipzig, Germany; 4 Department of Geography, Planning and International Development Studies and Centre for Sustainable Development Studies, University of Amsterdam, Amsterdam, the Netherlands; 5 Department of Agricultural Economics, Humboldt University of Berlin, 10099 Berlin, Germany

**Keywords:** Perceptions, Landscape dynamics, Participatory scenario development, GISCAME modeling, Integrated landscape approaches, Ghana

## Abstract

Settlement expansion and commercial agriculture affect landscape sustainability and ecosystem service provision. Integrated landscape approaches are promoted to negotiate trade-offs between competing land uses and their reconciliation. Incorporating local perceptions of landscape dynamics as basis for such negotiations is particularly relevant for sub-Saharan Africa, where most people depend on natural ecosystems for livelihoods and well-being. This study applied participatory scenario building and spatially explicit simulation to unravel perceptions of the potential impact of rubber and settlement expansion on the provision of selected ecosystem services in southwestern Ghana under a business-as-usual scenario. We collected data in workshops and expert surveys on locally relevant ecosystem services, their indicator values, and the probable land-use transitions. The data was translated into an assessment matrix and integrated into a spatially explicit modeling platform, allowing visualization and comparison of the impact on ecosystem service provision of land-use scenarios under rubber plantation and settlement expansion. The results show the capacity of current (2020) and future land-use patterns to provide locally relevant ecosystem services, indicating a decline in capacity of ecosystem service provisioning in the future compared to the 2020 land-use patterns, a threat to the benefits humans derive from ecosystems. This highlights urgent need for policies and measures to control the drivers of land-use/land-cover change. Furthermore, the results emphasize the importance of diversifying land-use/land-cover types for sustainable landscape development. The paper contributes new insights into how spatially explicit and semi-quantitative methods can make stakeholder perceptions of landscape dynamics explicit as a basis for implementing integrated landscape approaches.

## Introduction

Natural ecosystems provide diverse ecosystem services (ESs)—also referred to as nature’s contributions to people – essential to human livelihood, survival, and well-being (Díaz et al. 2018; Kadykalo et al. 2019). Some ecosystem services exert direct and tangible effects on human well-being, comprising regulatory services like climate regulation and water purification, provisioning services such as the supply of food, water, and fuel, as well as cultural services that encompass recreation and aesthetic beauty. Conversely, supporting services like soil formation and erosion control exert a more indirect yet foundational influence on ecosystem functionality and human welfare. These services collectively underpin the intricate relationship between ecosystems and the benefits they provide to societies. Recent studies continue to emphasize the criticality of understanding and preserving these multifaceted ecosystem services for sustainable development and human prosperity (Costanza et al. 2017; IPBES 2019; Torres et al. 2021; Hariram et al. 2023). Nonetheless, the integrity, status, and provisioning capacity of ecosystems are heavily influenced by land-use/land-cover changes (LULCC) (Gomes et al. 2021; Fang et al. 2022). Globalization and market demand for commodities have created opportunities for individuals and societies to alter land-use patterns (Kanianska 2016; Dieppe et al. 2018; Liao et al. 2023; Kayani et al. 2023). The pace, intensity, and extent of altering ecosystems for food, feed, and fiber are more intense now than observed in the past (Newbold et al. 2015; Ramankutty et al. 2018; Purswani et al. 2020; Achieng et al. 2023).

In 2006, a report published by the Millennium Ecosystem Assessment (MEA. 2005) revealed that 60% of the world’s ecosystem services (15 out of 24) were degraded or ineffective. According to the 2019 Global Assessment Report of the Intergovernmental Science-Policy Platform on Biodiversity and Ecosystem Services (IPBES 2019), 78% of the benefits humans obtain from nature (14 out of 18 categories) are rapidly declining, and land use, population, economy, technology, and other human activities are key contributing factors (IPBES 2019). Humans drive LULCC from the local to the global level through land-use decisions, exacerbating environmental degradation, climate change impacts, and ES provision. The causes and effects of LULCC should be understood as social-ecological processes affecting landscape sustainability (Magliocca et al. 2015; Verburg et al. 2015; Ren et al. 2019).

LULCC has received considerable attention in the landscape sustainability agenda due to the potentially negative consequences of LULCC for ecosystem services (Magliocca et al. 2015). For instance, The IPBES report (2019) highlights the risks of biodiversity and ES losses due to LULCC in Africa, where ESs are either poorly or unregulated (IPBES 2019). Specifically in Ghana, urban expansion resulted in a decline in the natural environment. For instance, in Accra, urban green spaces declined from 41% to 15% between 1991 and 2018, leading to a decline in ESs such as carbon storage, runoff, and regulation of soil quality (Puplampu and Boafo 2021). This disruption of ecosystem services caused by LULCC requires identifying context-specific causes of LULCC and addressing sustainability challenges related to land management, biodiversity, and ES provisioning (Meyfroidt et al. 2018). Therefore, studies on how land-use changes affect the ecosystems and their ES provisioning capacity are essential (Rounsevell et al. 2012).

Considering recent calls for integrated landscape approaches that aim to reconcile potentially competing land uses by mobilizing stakeholders to negotiate trade-offs between land uses (Sayer et al. 2013; Milder et al. 2014; Arts et al. 2017; Reed et al. 2020; Pedroza-Arceo et al. 2022), unraveling stakeholder perceptions of landscape dynamics and LULCC is of key importance. Considering such local perceptions informs sustainable land management strategies and fosters inclusive decision-making processes that consider diverse perspectives and priorities in landscape planning and land-use/land-cover change (LULCC) assessments (Aggrey et al. 2021; Asubonteng et al. 2021; Somuah et al. 2021). Participatory scenario development is a method for visualizing and planning potential future land-use change scenarios. It frequently starts by sharing knowledge to reduce risk and envisioning more inclusive, sustainable paths for people, the environment, and the economy (Oteros-Rozas et al. 2015). Scenario development also offers a platform to develop potential solutions to address identified environmental problems, supporting joint decision-making processes (Kariuki et al. 2021).

In line with integrated landscape approaches, participatory scenario development advocates the inclusion and participation of multiple land users in shaping future scenarios, fostering collaboration, and considering diverse perspectives to create more resilient and equitable outcomes (Mallampalli et al. 2016; Davenport et al. 2019). The process requires diverse knowledge types (Yanou et al. 2023a, b), multi-actor and multi-sector negotiations (Van Oosten et al. 2014; Ros-Tonen et al. 2018), addressing communication gaps (Karrasch et al. 2017), and managing data scarcity (Wolff et al. 2017). Therefore, these methods are significant in countries where individuals exercise minimal influence in decision-making processes, particularly in authoritarian regimes with weak democratic structures (Forsyth and Springate-Baginski 2021). Such circumstances are prevalent across various countries in the African continent (EIU 2020). Involving stakeholders in scenario development research is anticipated to enhance political viability and gain broader public acceptance compared to scenarios driven solely by experts (Oteros-Rozas et al. 2015). Participatory scenario development offers the possibility to discuss diverse opinions and deliberate and negotiate on issues to reach a consensus (Johansson 2021; Bayala et al. 2023; Siangulube et al. 2023). Thus, participatory scenario development is a relevant tool for researchers to unravel context-specific information that helps address the complexities and uncertainties in land-use decision-making and forecasting environmental change (Pereira et al. 2019)). The participation of diverse stakeholders in scenario development contributes to the credibility, quality, relevance, and legitimacy of the scenarios, particularly when all participants understand the process and outputs and a sense of ownership exists (Davenport et al. 2019; Reid et al. 2016).

Recently, participatory scenario development has been applied in several studies that explored stakeholder perceptions of land-cover/land-use change and desired future landscapes (Asubonteng et al. 2021; Bayala et al. 2023; Siangulube et al. 2023). However, the integration of spatially explicit simulation of stakeholder perceptions of current and future land-use change scenarios and its likely impacts on ESs remains understudied, especially in sub-Saharan Africa, with the study of Koo et al. (2019) among the exceptions. Such a study is highly relevant for southwestern Ghana, where rich biodiversity, spatial heterogeneity, natural resource endowment, resource management conflicts, high urbanization, and expanding industrialization processes exist. In addition, spatially explicit simulations help to identify likely areas of critical change (Ren et al. 2019). Combining LULCC scenarios generated by actor perceptions with spatially explicit land-cover models provides a consistent, logical, transparent, and replicable framework for land-use planning and management (Nicholson et al. 2019).

Unfortunately, Ghana has been faced with numerous challenges of insufficient or low compliance levels with land-use planning and management requirements, specifically due to low participation of stakeholder groups, weak enforcement provisions, and limited measures taken to address the concerns of an increasingly dynamic society (Awuah and Hammond 2014; Awuah et al. 2014; Akaateba et al. 2018). In other studies in the Ghanaian context, land-use plans have been extensively criticized for being driven mainly by experts, with little or no focus on addressing stakeholder rights, interests, and claims (Poku-Boansi and Cobbinah 2018).

Given the critical role actor perceptions of landscape dynamics and participatory and spatial tools play in achieving sustainable and inclusive landscape governance (Ros-Tonen and Willemen 2021; Ros-Tonen et al. 2021) and considering the research gap in sub-Saharan Africa and Ghana, this study aims to apply land users’ collective perceptions to simulate the impact of future land-use change scenarios on the provision of locally relevant ESs in southwestern Ghana. The study landscape is known for expanding rubber plantations through out-grower schemes and settlement expansions resulting from oil discovery and mining development (Bugri and Yeboah 2017; Asante-Yeboah et al. 2022).

The study addresses four research questions: (i) What is the current capacity of the study landscape to provide ESs to local people? (ii) How does a business-as-usual (BAU) scenario affect the capacity of the study landscape to provide locally relevant ESs? (iii) What challenges do the land users perceive in the capacity of ES provisioning under a BAU scenario? and (iv) What actions are needed for sustainable landscape development? These sub-questions will be addressed in the four sub-sections of the results, after which the implications will be discussed. But first, we elaborate on the methods used for this study in the following section.

## Material and Methods

### Study Area

**Fig. 1:**
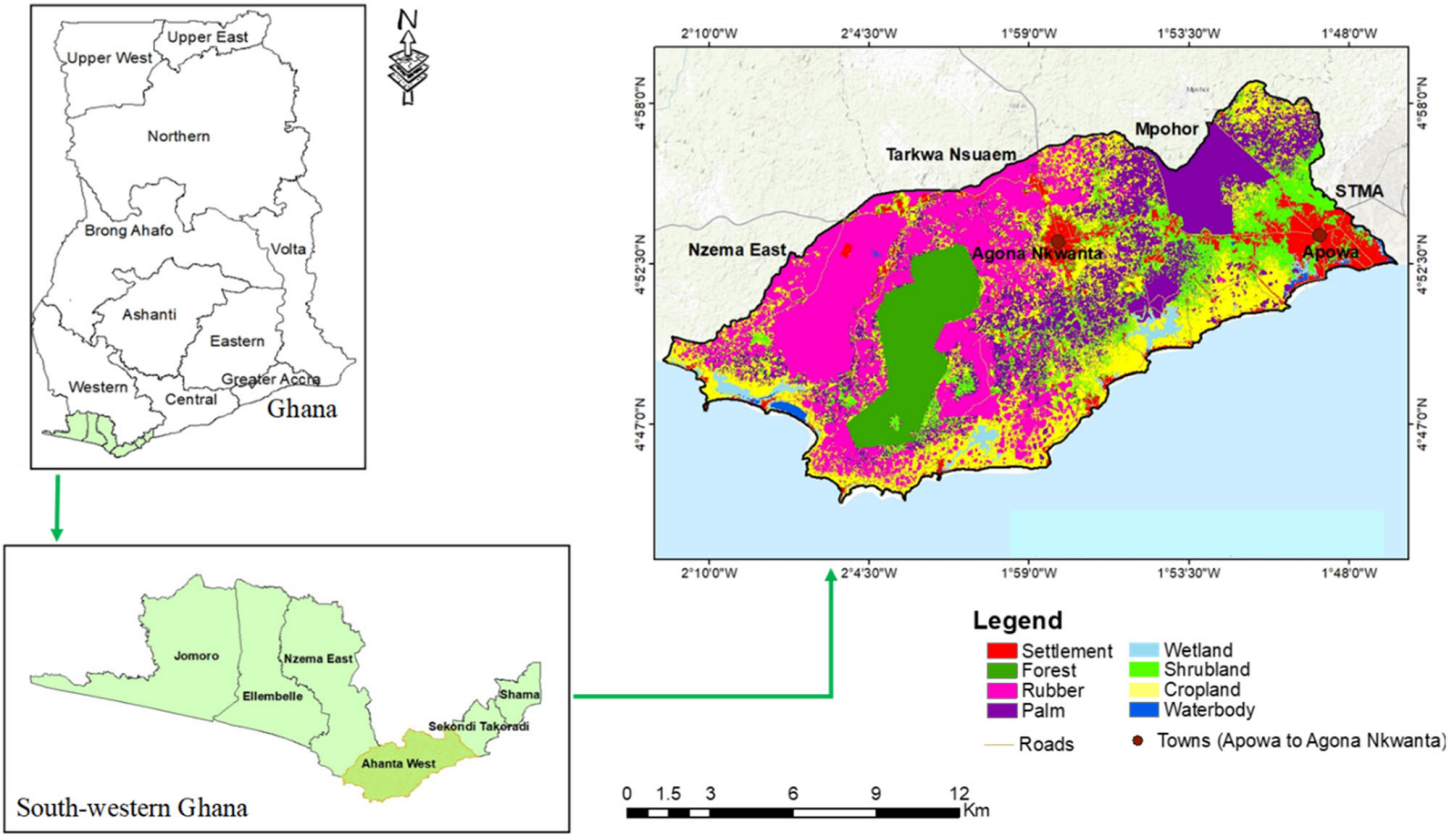
Land-cover map of Ahanta West Municipal Assembly in southwestern Ghana and location of Apowa and Agona Nkwanta. The land-cover map refers to the situation in 2020. Source: Asante-Yeboah et al. (2022)

The study area is the Ahanta West Municipal Assembly (AWMA), located in the southernmost part of Ghana between latitude 4°45′00″ N and 4°57′00″ N and longitude 1°45′00″ W and 2°13′00″ W (Fig. 1). AWMA borders the Gulf of Guinea in the south, the oil city Sekondi–Takoradi Metropolitan Assembly (STMA) to the east, Nzema East to the west, and Mpohor Wassa East District to the north. It covers an area of 591 km^2^ and has a total population of 138,192 (GSS 2019). In AWMA, most of the land is flat and covered with tropical rainforest vegetation. AWMA is one of the wettest places in Ghana, with a bimodal rainfall pattern: wet and dry seasons (AWMA 2018). The region’s dendritic drainage pattern, rich natural resources, exploitable oil reserves, and associated onshore infrastructural development favored economic development but have also put pressure on the land and natural environment (Bugri and Yeboah 2017; Otchere-Darko and Ovadia 2020) The region’s expanding rubber and oil palm industry and replacement of natural habitat with mono-cropping fields and infrastructure in the coastal landscape to meet economic and social demands potentially threaten biodiversity and ecosystem functions (deGraft-Johnson et al. 2010). However, ESs and landscape sustainability have rarely been considered in Ghanaian spatial planning and development programs (Inkoom et al. 2017).

### The Integrative Assessment Framework

**Fig. 2:**
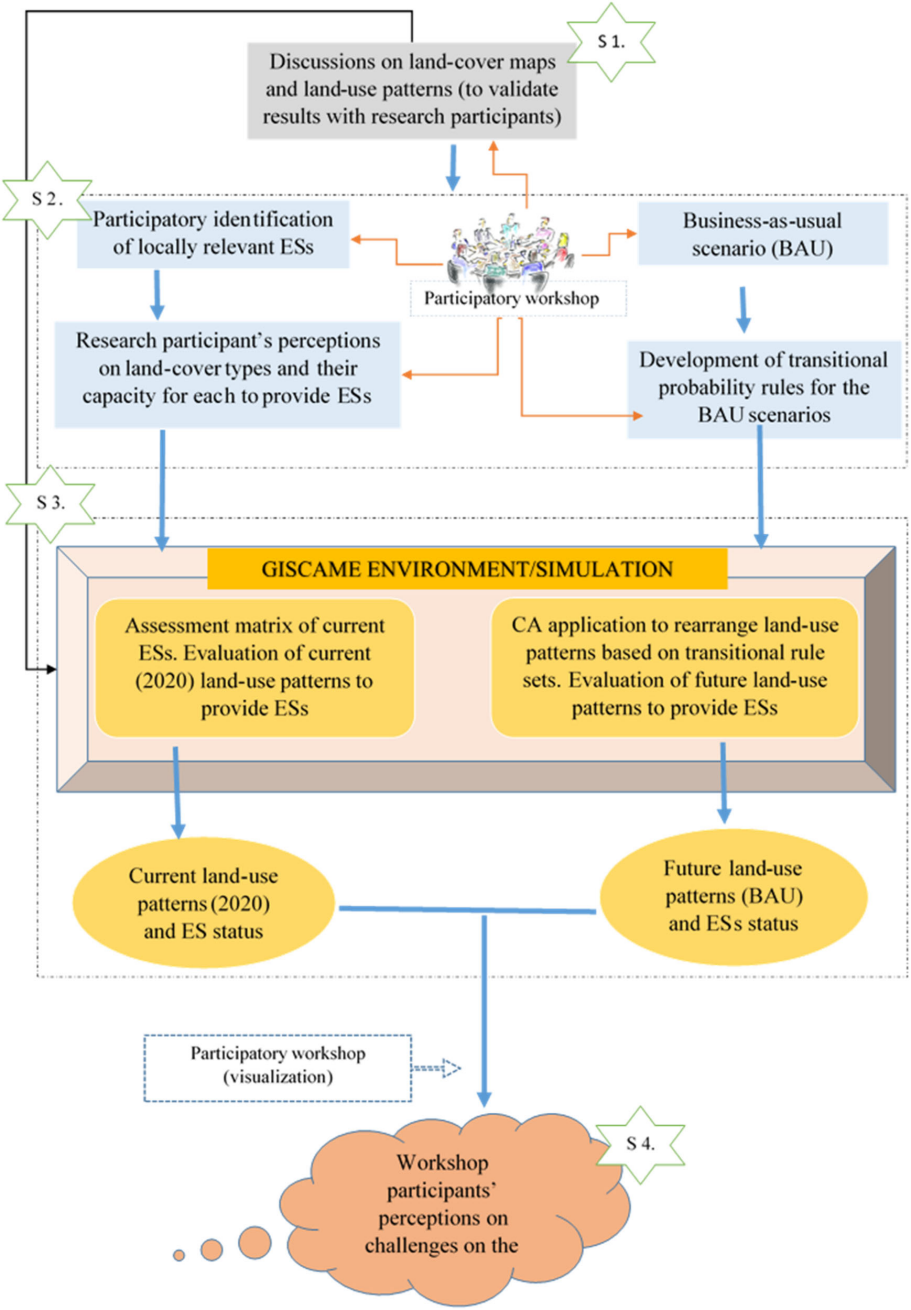
Visualization of the methodological framework applied in the study. The shapes on the left side in each step are the assessments of current land-use patterns (2020), and the shapes on the right are the assessments for future land-use patterns (under a BAU scenario). GISCAME means GIS geographic information system, CA cellular automaton, ME multi-criteria evaluation. Source: Authors’ construct based on visualization of the methodological flow of the study

This study employed a participatory scenario-building and spatially explicit simulation in four steps, visualized in Fig. 2. Step 1 (S1) comprises a discussion of the output of the four land-use/land-cover maps produced for the years 1986, 2002, 2015, and 2020. We discussed and validated the land-cover maps with research participants to ensure their local acceptance. Step 2 (S2) identifies the locally relevant ESs and captures research participants’ perceptions of the current land-use activities and their capacity to ESs. In Step 2, we also identified participants’ perceptions of plausible future land-use scenarios through transitional probability rules and participatory simulations. Step 3 (S3) is the data analysis part, which assesses the impact of future land-use scenarios on land-use patterns and ES provisioning. In this step, the collected local knowledge was combined with spatial data in a web-based simulation platform called GISCAME (GIS geographic information system, CA cellular automaton, ME multi-criteria evaluation), which is a landscape planning modeling software that can analyze how land management decisions can affect the capacity of ES provisioning and landscape functions using a perception-based approach (Fürst et al. 2010; Koschke et al. 2012). Finally, in Step 4 (S4), the research participants discussed and deliberated on the outcomes of the spatial simulations.

The study involved direct land users and institutional and industrial actors and captured their collective perceptions of the current and anticipated future state of the landscape (Villamor et al. 2014; Allan et al. 2022) in two workshops held between March and May 2021 (see workshop protocol outline in Supplementary Material 1). We applied stratified purposeful sampling to include only participants with a direct interest in using the land-use/land-cover types in the study landscape. Next, in consultation with the respective head or leader, we selected at least two persons per actor group for participation in the workshops, considering their knowledge and interest in land-use activities. Six farmers, two chiefs, eight institutional actors, and five industrial actors participated in the workshop (see Supplementary Material 2 for a description of the research participants).

The study focused on two drivers of land-use change in the study landscape, rubber and settlement expansion, to create a plausible future landscape under a BAU scenario. We chose these two for three reasons. First, the land-use/land-cover map of Asante-Yeboah et al. (2022) shows higher rubber and settlement expansion rates than other land-use/land-cover types in the study landscape. Second, the research participants recognized rubber and settlement expansion as the most significant drivers of land-use change in the study landscape, and third, the prevailing market conditions underlying rubber and settlement expansion facilitate their continuous expansion.

### Current Status of Land Use and Ecosystem Services

#### Description of land-use/land-cover types

The primary input for the spatial simulation process was the categorical land-cover map of AWMA produced from satellite images captured in 2020 using GIS and remote sensing methodologies (Asante-Yeboah et al. 2022). The land-cover map comprised eight main land-use/land-cover types (Table 1). About 22.26% of the landscape’s surface area consists of cropland (Table 1), reflecting a smallholder-agrarian landscape related to people’s main livelihood. The primary farming type is mixed cropping, combining a staple crop, e.g., cassava, plantain, or yam, with green vegetables and legumes to meet household food demands and dietary needs. In addition, AWMA’s climatic condition and soil characteristics make it suitable for cultivating commercial crops such as rubber and oil palm. Rubber covered an area of 27.35%, while oil palm occupied an area of 19.67%.

**Table 1:** Description of land-use/land-cover types in the study area and land share

Land-use/land-cover types	Description	Percentage of area (%)
Settlement	Rural communities, residential areas, industrial areas, bare concrete grounds, roads, and other artificial structures	7.61
Rubber plantations	Rubber plantations, including out-grower schemes	27.35
Palm vegetation	Smallholder and large-scale oil palm plantations and coconut fields.	19.67
Cropland	Annual and biannual food-crop farms, such as plantain, cassava, cocoyam, and vegetables	22.26
Forest	Cape Three Points Forest Reserve	9.09
Shrubland	Woody vegetation, including open areas, bushes, and fallow lands.	11.46
Waterbody	Rivers and other watercourses	0.55
Wetlands	Wetlands with mangroves	2.00

Source: Authors’ construct based on land-cover types and land share (in percentage) from the land-cover map (Asante-Yeboah et al. 2022)

#### The status of ecosystem services

An initial set of ESs was selected from the Common International Classification of Ecosystem Services (CICES V5.1). The ESs aligned with the benefits and predominant uses of smallholder mosaic landscapes in developing countries (Supplementary Material 3). Then, research participants indicated ESs considered “locally relevant” and their indicator values in a stakeholder workshop (Step 1 (S1) in Fig. 2. The workshop began with an introductory presentation on the current state of the study landscape (Asante-Yeboah et al. 2022). Next, participants were tasked to identify the locally relevant and significant ESs that reflect their livelihood needs using a Likert scale of 0–5 (from 0 = not relevant at all to 5 = highly relevant) (see Supplementary Material 4). The final set of ESs used in this study were those with an average Likert scale value above 4 (Table 2).

**Table 2:** Final list of selected locally relevant ecosystem services, indicators and proxies, and data generation methods used in the study

Ecosystem service	Definition	Indicator/proxy	Data source	Reference
Food provision	Wild and cultivated plants and terrestrial and aquatic animals for human nutrition	The proportion of products used as food for human consumption (%)	Workshop	(Haines-Young and Potschin-Young 2018)
Marketable products^a^	Products used to generate household income	The proportion of products sold for income (%)	Workshop	(Koo et al. 2019)
Fuelwood provision	Products used for household energy/cooking	The proportion of products used as fuelwood (%)	Workshop	(Haines-Young and Potschin-Young, 2018; Schmidt et al. 2019; Koo et al. 2019)
Soil quality regulation	Litter production and decomposition process and effect on soil quality	The litter decomposition rate of the land-use/land-cover type (%)	Expert survey through the Delphi method	(Haines-Young and Potschin-Young 2018; N’Dri et al. 2018; Giweta 2020; Saj et al. 2021)
Species diversity^b^	The diversity of species and varieties enabling the provision of ecosystem services	Type of species and amount of varieties in each land-use/land-cover type (Shannon diversity index)	Secondary data, expert survey	(MEA. 2005; Omayio and Mzungu 2019)

^a^Marketable products are not listed as such in the CICES list but were added because the research participants considered them locally relevant

^b^Although biodiversity was included as an ecosystem service in the Millennium Ecosystem Assessment (MEA. 2005), neither biodiversity nor species diversity was included in the CICES list. However, it is included here for its positive influence on the provision of ESs (Liquete et al. 2016a, b)

Indicator values of the capacity of different land-use/land-cover types to provide ESs were derived through actors’ perceptions of the capacity of different land-use/land-cover types to provide each ES with a Likert scale of 0–5 (from 0 = no capacity to 5 = very high capacity) (see Supplementary Material 5). Regarding ESs directly obtained from land-use/land-cover types as tangible benefits, e.g., food, fuelwood, and marketable products, identifying indicators for the ESs using local perceptions was feasible. However, using local perceptions, indicator values for two indirect ESs (species diversity and soil quality regulation) were hard to capture. For these ESs, we applied Shannon Wiener diversity index to calculate indicator values for species diversity and expert survey using the Delphi method to estimate the indicator/proxy values for soil quality regulation. The Delphi method is proper when there is a huge demand for time to collect sufficient field data (Walters et al. 2021). In this study, we engaged six experts knowledgeable about the characteristics of the ecological zone and land-use/land-cover types of the study area to generate a proxy value for soil quality regulation. The steps involved in the Delphi method are outlined in Supplementary Material 6. Species diversity/biodiversity was estimated using the Shannon-Wiener diversity index with a sample count of species from each land-use/land-cover type. The Shannon-Wiener diversity index is widely used in environmental studies, especially for simultaneously comparing two or more ecosystems (Omayio and Mzungu 2019). The species data was obtained from the organizations responsible for the identified land-use/land-cover types (e.g., Ghana Rubber Estates Limited (GREL) for the rubber plantation firm, Normpalm for the oil palm industry, The Department of Food and Agriculture (DoFA) for cropland, the Forestry Commission for the forest, and the district Physical Planning Department for open spaces).

### Development of Future Land-Use Scenarios and Simulation Conditions

This study focused on perception-based forecasting and spatially explicit simulations based on the collected information through a scenario-building workshop. Participatory forecasting evaluates the current conditions and predicts the likely future without intervention (Petropoulos et al. 2022). The workshop engaged participants in discussing the plausible future landscape scenario under a business-as-usual (BAU) trajectory. Expanding rubber plantations and settlements were considered the key drivers for LULCC under a BAU scenario. The local perceptions were specifically collected to elaborate spatial transition rule sets for simulating scenarios (Step 2 (S2) in Fig. 2). For instance, the participants were asked about the probability (%) of the change from one land-use/land-cover type to a rubber plantation or settlement, considering the current state of land use and without policy intervention. A Likert scale of 0–100 (75–100%: extremely probable, 51–74%: very probable, 31–50%: probable, 11–30%: not so probable, 0–10%: not probable) was used to identify the likelihood of individual conversion from current a land-use/land-cover type to rubber or settlement-related land-use/land-cover types (see Supplementary Material 7). In addition, neighboring land-use/land-cover types were discussed, which helped consider proximity effects influencing land-use changes.

### Data Analysis (Impact Assessment of Future Land-Use Scenarios)

We performed the simulation and scenario impact analyses using the GISCAME modeling platform (step 3 (S3) in Fig. 2). GISCAME is an effective tool for visualizing ESs provided by current and simulated land-use patterns and comparing scenario impacts, and the results are visualized as trade-offs or synergies between ES options (Fürst et al. 2011, 2013; Koschke et al. 2012; Koo et al. 2019).

We standardized the indicator values provided by the participants for each land-use/land-cover type with values ranging between 0 (the minimal potential of a land-use/land-cover type to provide the relevant ES) to 100 (the maximum potential of a land-use/land-cover type to provide the relevant ES) (Fürst et al. 2011; Koschke et al. 2012; Koo et al. 2018, 2019). The standardized values comprised an ES assessment matrix that presents the relationship between land-use/land-cover types and their capacity to provide ESs with the same value unit. We then simulated the BAU trajectory-based scenarios using the CA module embedded in GISCAME. CA is a discrete dynamic cell base system that converts the state of a cell based on a rule set regarding its neighboring cells and its own environmental status (Koschke et al. 2013; Koo et al. 2018). The transition rule sets can be iteratively applied in the GISCAME platform for many time steps to show the impacts of temporal or intensification of land-use changes. In this study, we agreed with the participants to apply the transitional probability rule set as one iteration to simulate five years from the current state (year 2020) and ten iterations to simulate 50 years into the future from the current state (year 2020).

Newly generated land-use patterns by the CA process were combined with the ES assessment matrix to show the capacity of the study area to provide ESs. The final assessment score indicates mean values for the ESs provided by individual land-use cells within the area (Koo et al. 2019). The spider chart, the ES provisioning map, and the ES balance table represented the final outputs. By comparing the mean ES values provided by current and future land-use patterns, potential trade-offs or synergies between ESs caused by land-use changes at the landscape level were identified. The assessed results were shared and discussed with the participants in another workshop (Step 4 (S4) in Fig. 2), Supplementary Material 1). The participants gave us their feedback on the simulated results, the anticipated challenges of the plausible future landscape, and the needed measures toward the sustainability of the landscape.

## Results

### Capacity of Land-Use/Land-Cover Types to Provide Ecosystem Services

Table 3 presents the values of locally relevant ESs provided per land-use/land-cover type: food, marketable products, fuelwood, species diversity, and soil quality regulation. Cropland showed the highest capacity to provide food. Additionally, the research participants considered fruit trees and bushmeat from forest and shrubland, oil palm and coconut fruits from palm vegetation, and aquatic food from wetland and water bodies to contribute to the food products; hence, these land-use/land-cover types were assigned values under food provisioning. Settlements and rubber plantations showed no capacity for food provisioning. The landscape capacity to provide marketable products was mainly delivered by rubber plantations, followed by palm vegetation and cropland (Table 3). The research participants also considered the collection of firewood and other products (fruits, aquatic food, non-timber forest products) from forests and wetlands as marketable products (Table 3). Research participants consider mangroves (within wetlands) as the primary source of fuelwood for smoking fish, the main non-farming livelihood in this landscape. They also identified tree branches from cropland, palm vegetation, shrubland, and forests; pruned branches from rubber plantations; and stalks of crops as sources of fuelwood for household cooking and energy. The research participants perceived the forest to provide substantial species diversity, followed by cropland. The habitat provided by mangroves for the proliferation of other species, palm intercropping with other crops, and shrubland were also considered to contribute to species diversity. Settlements and rubber plantations showed no capacity to provide species diversity. Lastly, cropland presented the highest capacity for the regulation of soil quality. The other land-use/land-cover types showed moderate levels to regulate soil quality except for settlements and water bodies.

**Table 3:** Ecosystem service assessment matrix showing the relationship between land-use/land-cover types and their capacity to provide ESs within a range between 0 (no capacity to provide ESs, in white) and 100 (highest capacity to provide ESs, in dark purple)

Source: Authors’ construct based on the normalized ES values.

**Fig. 3:**
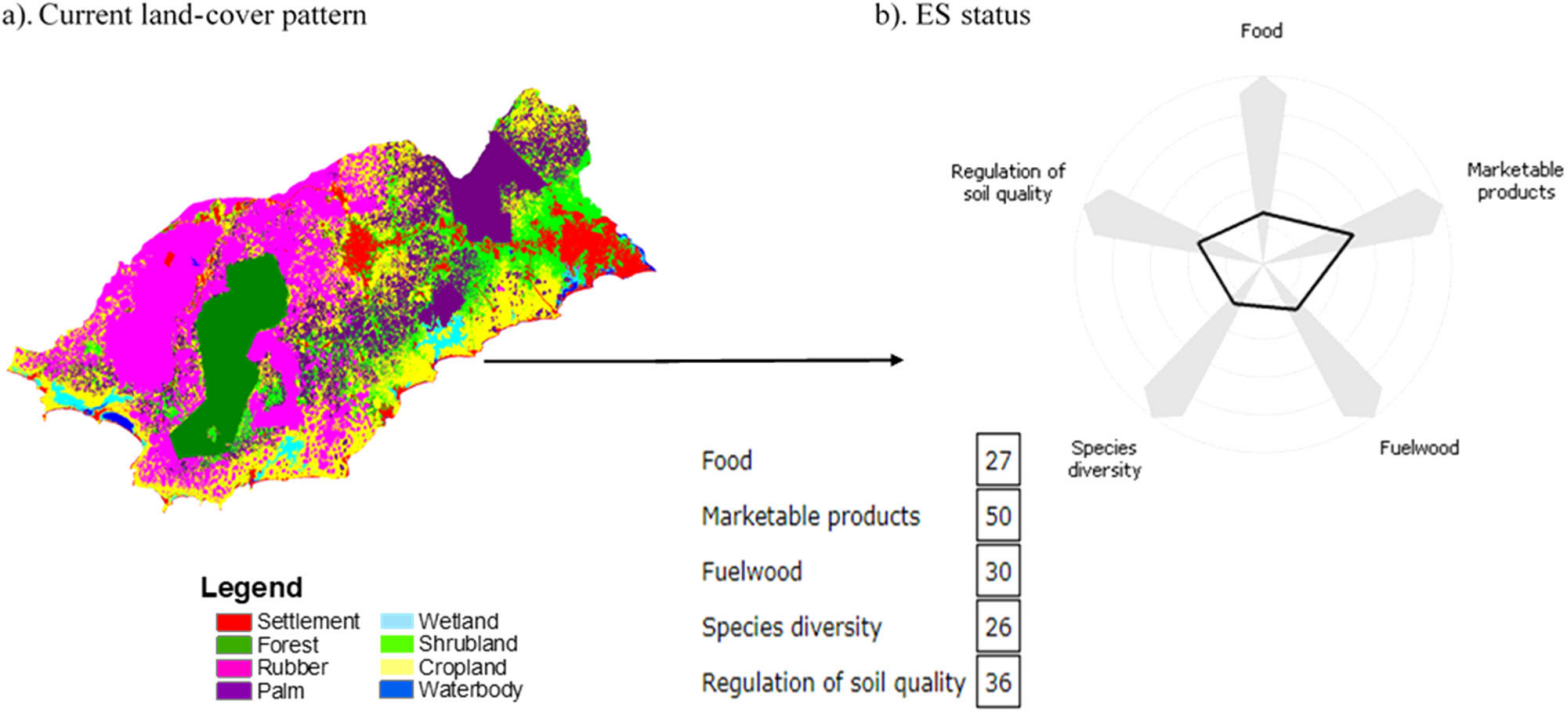
Ecosystem service values at the landscape level: **a** land-use pattern (2020), **b** Spider chart showing the ecosystem service status under the current land-use pattern. Source: Authors’ construct based on the simulation of the land-use pattern (2020) in the GISCAME environment

The assessment matrix (Table 3) links the current capacity to provide ESs to the land-use/land-cover types from Fig. 1, which leads to Fig. 3. The ES balance tables illustrate the individual ES values corresponding to the spider chart. Marketable products exhibit the highest ES value in the study landscape, followed by soil quality regulation, while the landscapes’ capacity for species diversity provision was the lowest. Food provision was the second lowest and slightly higher than species diversity provision.

### The Impact of Land-Use Change Scenarios on the Provision of Ecosystem Services

The transition rule set for simulating rubber and settlement expansion is shown in Table 4. Neighboring conditions indicate proximity effects at the landscape level, such as geographical location or neighboring land-use/land-cover types. This means that depending on the geographical location of a land-use/land-cover type, either rubber or settlement expansion can occur, but not simultaneously. Regarding rubber expansion, the research participants perceived cropland, shrubland, and palm vegetation as potential land-use/land-cover types to be converted to rubber plantations with high transition probabilities of approximately 85%, 90%, and 60%, respectively (Table 4). The research participants indicated location in the western part of the study area (rural areas) and neighboring land uses such as cropland, shrubland, and palm vegetation as neighboring conditions for the conversion to rubber plantations (Table 4). Forest, wetland, and settlements in the western part of the study landscape showed a lower likelihood of rubber expansion in the perception of the research participants. Regarding settlement expansion, the research participants perceived cropland, shrubland, and palm vegetation to have a transition probability to settlement of approximately 50%, 90%, and 50%, respectively. They also perceived settlement expansions in settlement areas to be more likely to concentrate in the eastern part of the study landscape (peri-urban areas).

**Table 4:** Transition probabilities (in percentages) of land-use/land-cover types and neighboring conditions associated with each land-use change driver

Land-use driver	Initial land-use/land-cover type	Target land-use/land-cover type	Transitional probability (%)	Neighboring conditions
Rubber expansion	Cropland Shrubland Palm vegetation	Rubber plantation	85 90 60	Location of the initial land-use/land-cover type in the western part of the study landscape. The proximity of the initial land-use/land-cover type (cropland, shrubland, or palm vegetation) to a rubber plantation, with or without one cell of rubber plantation located as neighboring cells around cropland, oil palm, rubber plantations, and shrubland.
Settlement expansion	Cropland Shrubland Palm vegetation	Settlement	50 90 50	Location of the initial land-use/land-cover type in the eastern part of the study area and along the 15-km road stretch from Agona to Apowa cape (Fig. 1) (Bugri and Yeboah 2017). The proximity of the initial land-use/land-cover type to the major road network. With or without one cell of settlement as neighboring cells to shrubland, cropland, or palm vegetation.

Source: Authors’ construct based on discussions with research participants.

#### The impact of rubber expansion

**Fig. 4:**
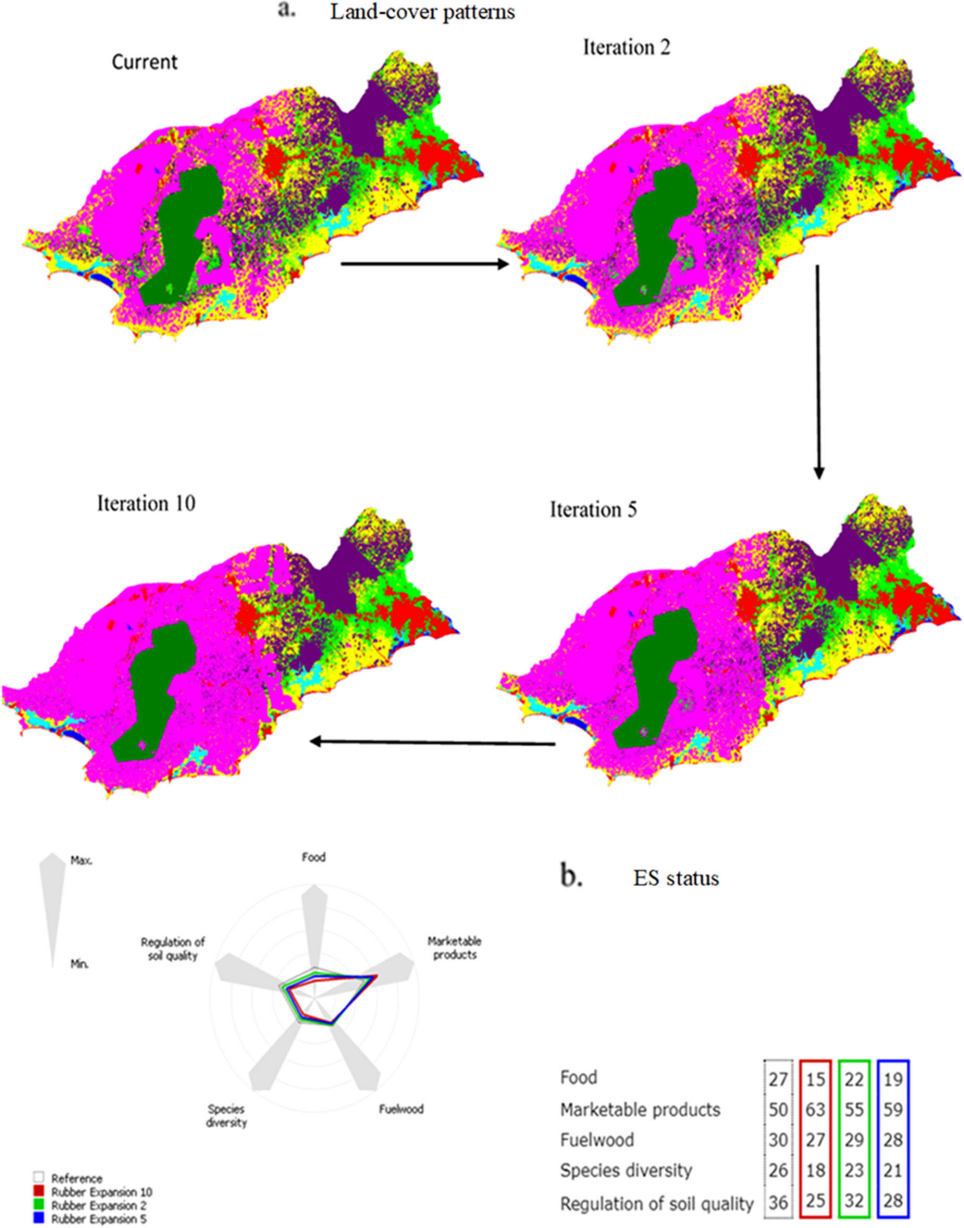
Impact of rubber expansion on land-use pattern and ecosystem service provisioning in the study region. The spider chart displays the change from the current (2020) ecosystem services provisioning level (gray color line) to a future state by intensifying rubber cultivation. As agreed with the participants, the green line means a two-time iteration simulation (Rubber expansion 2), which equals ten years; the blue line a five-time iteration simulation (Rubber expansion 5), which equals 25 years; and the red line denotes a ten-time iteration simulation (Rubber expansion 10), which equals 50 years. The table on the right side of the spider diagram corresponds to the spider chart, which indicates the landscape capacity of ecosystem service provisioning. Source: Authors’ construct based on the GISCAME simulation under rubber expansion

Applying the transition probability rule set (Table 4; Supplementary Material 7), the conversion to rubber plantations resulted in a negative area change in most land-use/land-cover types (Table 5). The analysis identified a considerable conversion from oil palm plantations, shrubland, and cropland to rubber plantations (Table 5). The iterative application of the transition rule set revealed the intensification of rubber expansion (the expansion of pink areas in the land-use/land-cover maps, Fig. 4) and visualized the trade-offs between rubber plantations and other land-use/land-cover types (Table 5). However, the impact of rubber expansion on settlements, forest, wetland, and water bodies was insignificant (Table 5). Rubber expansion similarly decreased the ES values, especially food provision and regulation of soil quality (Fig. 4). Conversely, marketable products were increased as a trade-off (Table 5).

**Table 5:** Area change of land-use/land-cover types influenced by the intensification of rubber expansion

Land-use change	Land-use/land-cover type	Iteration 2 (%)	Iteration 5 (%)	Iteration 10 (%)
Rubber expansion	Settlement	0	−0.01	−0.02
	Forest	0	0	0
	Rubber plantation	8.33	14.69	21.19
	Palm vegetation	−2.56	−4.58	−6.56
	Wetland	0	0	−0.01
	Shrubland	−1.48	−2.43	−3.23
	Cropland	−4.29	−7.66	−11.37
	Waterbody	0	0	0

Source: Authors’ construct based on the difference between the simulated iterations and the current year (2020) under rubber plantation simulation.

#### The impact of settlement expansion

**Fig. 5:**
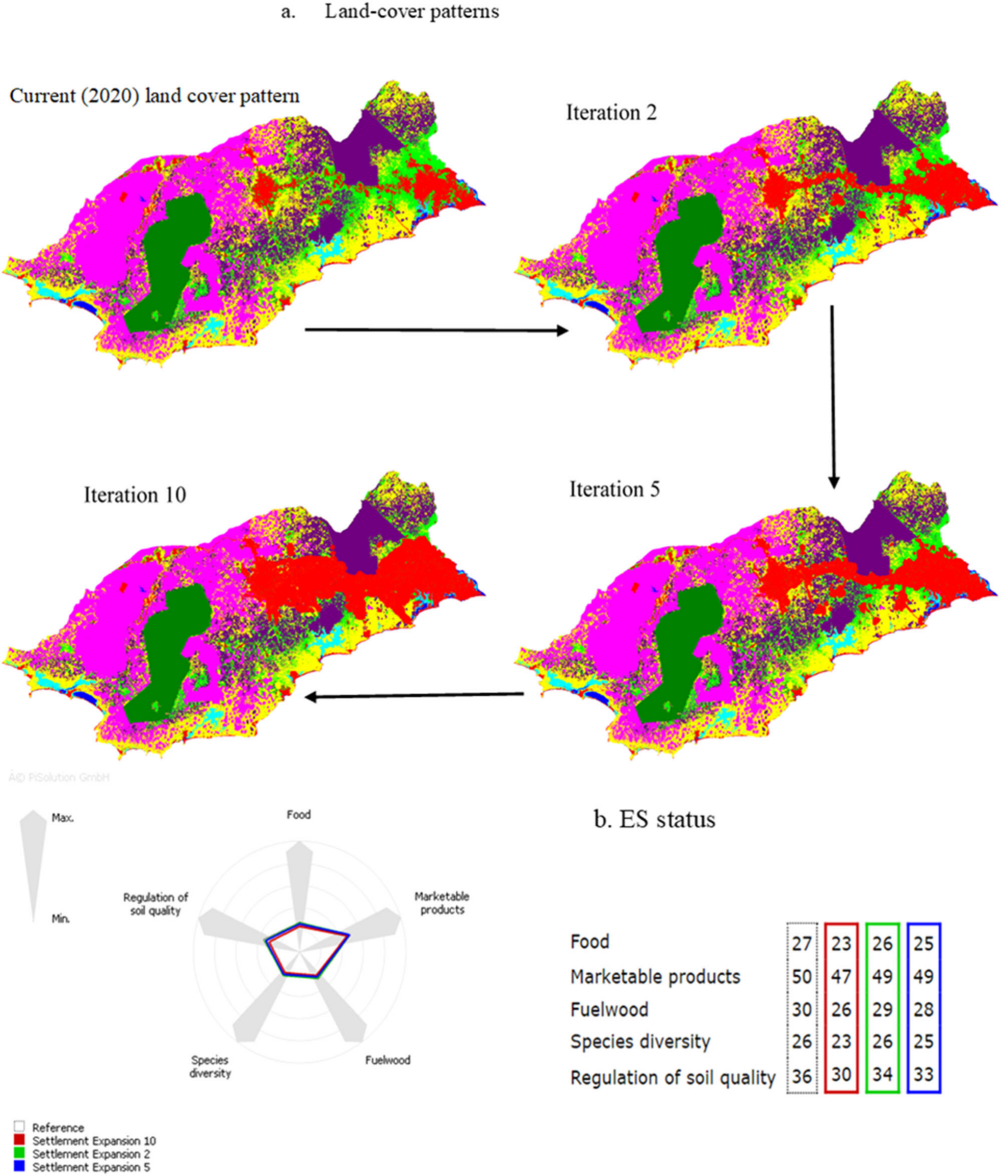
The impact of settlement expansion on land-use patterns and ecosystem service provisioning in the study area. The spider chart displays the change from the current (2020) ecosystem services provisioning level (gray color line) to a future state as settlements expand. As agreed with the participants, a two-time iteration simulation (Settlement expansion 2) equals ten years (green line); the blue line means a five-time iteration simulation (Settlement expansion 5) equaling 25 years; and rubber expansion 10 equals 50 years and is denoted by red lines. The table accompanying the spider chart indicates the landscape’s ES provisioning capacity

Settlement expansion resulted in a distributional change in the land-use/land-cover types (the expansion of red areas in the land-use/land-cover map, Fig. 5). Palm, shrubland, and cropland declined in area changes compared to the initial land-use/land-cover types, whereas settlement only increased (Table 6). The other land-use/land-cover types recorded insignificant changes in the area by all the iterations. Impacts on the landscape’s capacity to provide ESs under settlement expansion resulted in decreases in all the ESs provided by the landscape (Fig. 5).

**Table 6:** Area change of land-use/land-cover types influenced by settlement expansion

Land-use change	Land-use/land-cover type	Iteration 2 (%)	Iteration 5 (%)	Iteration 10 (%)
Settlement expansion	Settlement	2.99	5.55	11.09
	Forest	0	0	0
	Rubber	0	−0.01	−0.01
	Palm vegetation	−0.15	−0.46	−2.33
	Wetland	0	0	0
	Shrubland	−2.39	−3.79	−5.71
	Cropland	−0.45	−1.26	−3.02
	Waterbody	0	0	0

Source: Authors’ construct based on the difference between the simulated iterations and the current year (2020) under settlement expansion simulation.

### Challenges of the BAU Scenario

The research participants shared their views of potential social, economic, and environmental threats under the plausible future landscape BAU scenario. These included food shortage and reliance on the market for food supply, a change in cultural/traditional values, land degradation due to rubber expansion, species habitat degradation, ecosystem degradation, and reduction in species diversity (Table 7). One participant (farmer) mentioned:

‘Today, during farmers’ day celebration, the different types of crops displayed have reduced dramatically compared to about ten years ago. We used to have traditionally grown vegetables and crops, but in recent times, we no longer have them.’ (workshop participant Agona Nkwanta, May 2021).

**Table 7:** Participants’ views of environmental and socioeconomic threats under the ‘BAU’ scenario

Environmental and socioeconomic threats	Rubber expanding area	Settlement expanding area
Land degradation	✓	✓
Low soil fertility	✓	✓
High food prices	✓	✓
Food shortage	✓	✓
A decline in crop diversity	✓	✓
Loss of transitional landraces^a^ (e.g., yellow maize, local yam)	✓	✓
Loss of traditional/cultural values	✓	✓
Market price volatility	✓	✓
A decline in peri-urban farming		✓
Climate change impacts	✓	✓
Increase in poverty due to a drop in rubber prices	✓	
Species habitat destruction	✓	✓
Reduced pollination	✓	✓

Source: Compiled by the authors based on discussion with the research participants during the workshop

^a^Landraces are defined as “plant materials consisting of cultivated varieties that have evolved and may continue evolving, using conventional or modern breeding techniques, in traditional or new agricultural environments within a defined ecogeographical area and under the influence of local human culture” (Casañas et al. 2017, p. 2)

One participant from the institutional sector lamented the likely decline in rubber prices and its consequence on local farmers’ livelihoods and economic life. The farmers were alarmed by the implications of the possible decline in rubber prices. One participant (rubber farmer) expressed his fears as follows:

‘When rubber prices drop, we cannot sell the latex from our rubber farms. This means our land will be locked, and there will be no money from the sale of rubber and no land for food production. We, therefore, cannot purchase adequate food from the market for our household’ (workshop participant Agona Nkwanta, May 2021).

Participants from the Spatial Planning Department drew attention to settlement-expanding areas and the likely threat of food price volatility and competition with the oil industry for food items. In addition, peri-urban farming decline and land scarcity were perceived as additional challenges under the BAU scenario.

### Measures Towards Sustainable Landscape Development

‘Farmers are always excluded from land-use planning and zoning. But if we want our land-use plans to work effectively, all actors in the land-use sector should be consulted to develop our local land-use plans” (Farmer, Punpuni, AWMA, Agonal Nkwanta, May 2021).

‘The Physical Planning Department is mandated to prepare structural and local land-use plans. However, the unit does not have control over the specified usage of the land. Land ownership in Ghana is customary, and family heads/landowners can change the intended use of the land to another purpose, of which the Physical Planning Department has no power to revoke this’ (Physical planner, AWMA, Agona Nkwanta, May 2021)’

The research participants deliberated on land-use planning issues and considered the following three actions as key to ensuring sustainable land-use transformation and landscape development.

#### Sensitization

The trade-offs between land-use/land-cover types and ESs under the simulated BAU scenario suggested local ES degradation and livelihood implications. The research participants expected sensitization on the harmful impacts of current land-use practices to instigate behavioral change and shared support for reducing detrimental practices. Sensitization at different stakeholder levels and platforms, such as reaching the youth via schools and the general public via the media, churches, and handbills, was considered appropriate to raise awareness of landscape dynamics.

#### Development of alternative site-specific land-use scenarios

The research participants expected they could contribute to enhancing ESs by changing their land-use practices. They agreed that planning for a sustainable future will require the inclusion of all actors to discuss how trade-offs can be minimized, compromises can be made, and adverse land uses can be replaced with or compensated by sustainable land uses. Engaging local farmers, land owners/chiefs, plantation industries, real estate developers, and the District Assembly is key to the success of such planning.

#### Formulation of policies and laws

Designing policies backed by laws to govern the implementation of alternative land-use scenarios was recognized as vital in achieving landscape sustainability. Agricultural plantation schemes on out-growers schemes that do not advance multifunctionality should be revisited and revised to allow farmers to practice multifunctionality in land uses. Enforcing laws on land use towards sustainability should extend beyond electoral cycles, and sanctions should be applied to offenders. A bottom-up approach to developing laws and a highly consultative approach was considered more effective for adoption.

## Discussion

### Local Perceptions of Ecosystem Service Provisioning and Landscape Change

The participants perceived food, fuelwood, marketable products, soil quality regulation, and species diversity as the most locally relevant ESs in the study landscape (Table 2). The identified ESs are characteristic of smallholder landscapes in sub-Saharan Africa and are key to human well-being, contributing to poverty alleviation, climate mitigation, and economic resilience while delivering various goods and services such as food, water, biological diversity conservation, and soil quality regulation (Milder et al. 2014). The AWMA (2018) describes the study landscape as largely rural, with about 66% of the population dependent on smallholder agriculture. The agricultural activities and nature dependency may explain why the research participants considered these ESs, in particular, as locally relevant.

Furthermore, the participants regarded cropland as the land-use/land-cover type providing the most food but viewed rubber and palm plantations as the two land-use/land-cover types provisioning the most marketable products (Table 3). The recent shift in crop choices favoring rubber and (oil) palm shifts subsistence farming on cropland to commercial agriculture on rubber and palm plantations. The perception that wetland, including mangroves, ranks highest in fuelwood provisioning is because of the particular demand for mangrove wood for smoking fish, a common non-farming livelihood in the study landscape (Nunoo and Agyekumhene 2022). Mangrove wood is perceived to infuse an exceptional taste into smoked fish, attracting higher prices on the market (Jones et al. 2016).

Species diversity, a measure of species richness, abundance, and distribution, was estimated to be relatively higher in the forest than in other land-use/ land-cover types (Table 3). Participants agreed with the estimated indicator value for the forest and explained that the forest as a reserve is not accessible to the public; hence, human interference is limited. According to the forest and wildlife report of the Cape Three Points Forest Reserve, the recent ecosystem survey recorded 17 species of medium and large mammals, 27 tree species, and 45 species of butterflies (Hen Mpoano 2019). In Ghana, forest reserves and national parks cannot be used for agricultural purposes due to regulations and laws for their establishment and management. For example, the Forest Protection (Amendment 2002) Act 624 and the Forest Act 1927 (CAP 157) prohibit agricultural activities in forest reserves. Strong regulations and actions may have contributed to the assumption that forests are the land-use/land-cover type providing the highest species diversity.

The intercropping system practiced in the study landscape (where the main crop is interplanted with other minor crops) may explain why cropland ranks second in species diversity provision. Farming in the southern part of Ghana is more heterogeneous than the monocropping system in the northern parts of Ghana, favored by the bimodal rainfall pattern compared to the unimodal rainfall pattern in the North (Kuivanen et al. 2016; Bellon et al. 2020). Integrating different crop species in a mixed-cropping system enhances the resilience of farming and cushions farmers during periods of environmental shocks (Asfaw et al. 2019; Bellon et al. 2020).

Soil quality regulation, measured by litter fall and decomposition rate, was perceived to be the highest in cropland (Table 3). The experts in the Delphi method attributed this to the seasonal and perennial cropping system and land preparation methods locally known as *proka*. Under this system, harvested debris is left on the ground to rot and mix with the soil to facilitate high litter production and decomposition in cropland. Other studies in sub-Saharan Africa also mention cropland/agricultural lands as contributing to soil quality regulation (e.g., Brinkmann et al. 2019; Tiwari et al. 2019; Fenta et al. 2020; Muchane et al. 2020).

Interestingly, the participants perceived rubber expansion to occur mainly in the western part of the study landscape, while settlement expansion occurs in the eastern part. This corresponds with the land-cover/land-use map produced with GIS and remote sensing methodologies by Asante-Yeboah et al. (2022) that visualizes landscape change due to rubber and settlement expansion. The participants perceived rubber to dominate the western part of the study landscape and expected other land-cover shifts to rubber plantations to occur in this region. Conversely, the participants expected settlement expansion to occur mainly in the eastern part of the study landscape and along the road stretch between Agona Nkwanta and Apowa (Fig. 1). This perception corresponds with the satellite-based land-cover maps of the study landscape, which show that rubber expanded more than three times its initial size over the last 34 years in the western part of the study landscape, while settlements grew more than four times their initial size and dominated the eastern part (Asante-Yeboah et al. 2022). The participants attributed this to the availability of farmlands and the crop preferences of farmers/landowners in the western part of the study landscape. They also mentioned the lower economic returns from food-crop farming compared to the higher financial returns from rubber plantations as a reason for the shift from cropland to rubber plantations. These findings concur with those of Bugri & Yeboah (2017). Other factors, like the prevalence of customary land ownership in rural areas, may account for the easier conversion of farm and fallow lands into rubber plantations in these areas (Kasanga and Kotey 2001). This is more complicated in Ghana’s urban areas, where statutory land ownership prevails. This is also part of the explanation for participants’ perception that rubber expansion occurs mainly in the western, more rural part of the study landscape.

Regarding the eastern part of the study landscape, the participants also perceived the influence of oil discovery and related economic activities in the neighboring city, Sekendi-Takoradi, which persuades landowners to release lands for infrastructural purposes. In addition, the road network, accessibility, and improved infrastructure facilitate the ‘hot spot’ settlement expansion along the 15-km road stretch from Agona Nkwanta to Apowa (Fig. 1). The influx of migrants and the presence of international oil companies in Sekendi-Takoradi, well-known as the oil city of Ghana, are driving investments in commercial development and real estate in this region (Fiave 2017). The oil city is expanding horizontally toward outlying towns, resulting in infrastructural development, building construction, and settlement expansion in the eastern part of the study landscape (Obeng-Odoom 2014; Mensah et al. 2018). Surprisingly, the participants did not mention environmental conditions that influence the land-use/land-cover changes to either rubber plantation or settlement. For example, rubber establishment requires a slope of less than 20% (pers. comm. GREL Officer, AWMA, March 2021). The study landscape falls below this slope category; hence, converting land into rubber plantations is not hindered by environmental attributes. Structural plans prepared for the study landscape broadly categorize about 50% of the eastern part of the landscape as suitable for infrastructure; hence, environmental attributes did not significantly affect the land-cover change (pers. comm. Physical Planning Department, AWMA, March 2021).

### Strengths and Weaknesses of the Methodology

This study used qualitative data on the perceptions of research participants in a quantitative modeling framework to generate site-specific spatially explicit information vital to conservation policies and sustainable use of natural resources and ecosystems (Beck et al. 2014). The participatory approach adopted in this study to assess and interpret the relationship between land-use/land-cover types and their capacity to provide ESs differs from the scientifically oriented viewpoint as applied in other studies (Vrebos et al. 2015; Anderson et al. 2017; Phillips and João 2017; Arowolo et al. 2018). In this participatory approach, we identified the locally specific challenges that may be associated with future landscape change and the possible measures to ensure the sustainability of the landscape. The participatory approach and inclusion of local perceptions allowed for a reflection on local people’s experiences, which have evolved through trial and error and have proven flexible enough to cope with change (Feurer et al. 2019; Johansson 2021). Such local knowledge complements existing scientific-based findings (e.g., Kettle et al. 2014; Posner et al. 2016; Klenk et al. 2017). The participatory approach also strengthens the unraveling of local ESs provided by different land-use/land-cover types. In so doing, we could capture the multiple benefits of each land-use/land-cover type. For example, in identifying food provision, the study captured all components of a land-use/land-cover type used as food for household consumption. The approach allowed expressing indicator values in percentages rather than limiting food provisioning to only yield per hectare as used in other ES studies (e.g., Dunford et al. 2015; Palacios-Agundez et al. 2015; Czúcz et al. 2018; Bethwell et al. 2021). Using yield per ha only assumes a single ES is derived from a land-use/land-cover type (e.g., woodlot only provides fuelwood, cropland only provides food, and monocropping farms only provide marketable products) (Li et al. 2017). In addition, using a participatory approach, we could eliminate double counting, such as valuing fuelwood for both household consumption and a marketable product (Koo et al. 2019). A participatory approach that involves farmers and local land users in land-use governance increases participation and local negotiation power in decision-making (Asubonteng et al. 2021).

However, the approach exhibited some weaknesses that allowed the exclusion of some important ESs, such as pollination, carbon sequestration, flood control, and aesthetic beauty (recreation and intrinsic value), which are prominent ESs in the study landscape. However, indicators to assess these ESs were challenging. First, capturing and assessing ESs, such as aesthetic values captured at the landscape scale or in a regional assessment, requires an analysis of the structural dynamics of the landscape (Frank et al. 2013; Inkoom et al. 2018). A perception-based approach, as applied in this study, based on collective perceptions, may require the application of additional methods to compare the opinions of different groups of research participants and individuals as aesthetic and other intangible values are subjective. Other intangible ESs, such as pollination, flood control, and carbon sequestration, were difficult to explain to the research participants, and assessing their perceptions of these intangible ESs was, therefore, not feasible. Other approaches, such as field data collection, quantification, laboratory analysis, and expert judgment, could have been used to obtain ES indicator values or proxies for some of these intangible ESs, but this was beyond the scope of this study due to cost and time restrictions, and lack of experts. Therefore, the study acknowledges that the perception-based approach limits the ability to identify all relevant ESs in a local context. However, the perception-based approach helps unravel local knowledge of ESs and raise awareness of the adverse effects of landscape dynamics on ES provisioning, which contributes to effective site-specific development of land-use policies to advance landscape sustainability (c.f. Asubonteng et al. 2020).

### Implications of Land-Cover/Land-Use Change on Landscape and Ecosystem Service Provision

The results of the participatory scenario building and spatially explicit simulation indicate risks regarding landscape sustainability and management. For instance, in the eastern part of the study landscape, where settlement is expanding, the population consists primarily of skilled workers who have access to various employment opportunities and diversified income in the oil industry (Fiave 2017; Ablo 2018; Otchere-Darko and Ovadia 2020). However, most people residing in the western part of the study landscape are smallholder farmers and unskilled workers (Otchere-Darko and Ovadia 2020). Their livelihoods depend mainly on subsistence farming. The inter-cropping system in southern Ghana functions as a risk-copping strategy against poorly functioning markets. For example, aside from their cultural significance (Hoffmann and Gatobu 2014), diversified cropping systems reduce farmers’ vulnerability to market and climate variability (McCord et al. 2015) and contribute to household dietary diversity. According to the research participants, the disappearance of cropping diversity due to their replacement with rubber plantations and settlements will result in the erosion of the multifunctional capacity of the landscape.

The results indicate the need for urgent actions for balanced and sustainable ES provision in the study landscape. Agriculture should be practiced more sustainably, for instance, by adopting basic land and soil management to improve the quality of agricultural lands (Haregeweyn et al. 2023) and applying climate-smart agriculture (see, e.g., Lipper et al. 2014; Chandra et al. 2018). Adopting the urban densification concept, which entails strategically increasing the population and built environment density within existing urban areas, aims to optimize land-use efficiency, enhance infrastructure utilization, and promote sustainable urban growth. This may reduce the intensity of land-cover change to settlement (Peng et al. 2017).

## Conclusion

This study applied a semi-quantitative method by combining participatory scenario building and a spatially explicit simulation approach to assess how land-use change driven by rubber and settlement expansion can impact ecosystem health in southwestern Ghana. By developing BAU scenarios, we established the impact of rubber and settlement expansion on the capacity of land-use/land-cover types to provide ESs that research participants considered locally relevant. The results showed trade-offs and ecosystem degradation with the expansion of rubber plantations and settlement. The scenario analysis showed that continuing the current pattern of land-use practices will lead to a decline in the ecosystem benefits for human well-being. Hence, we advocate adapting current land-use practices and reviewing land-use policy schemes. The approach adopted offers the possibility of integrating the perceptions of landscape actors in landscape decision-making to ensure land-use planning policies are feasible and acceptable. The authors argue that combining qualitative, quantitative, and spatial methods allows for incorporating local research participants’ rich and context-specific knowledge to generate insights into a plausible future state of the landscape. This is a prerequisite for developing much-needed policies for landscape sustainability.

### Supplementary information

Supplementary Materials
