# Supplementary material for: Participatory and Spatially Explicit Assessment to Envision the Future of Land-Use/Land-Cover Change Scenarios on Selected Ecosystem Services in Southwestern Ghana
Source: Environ Manage. 2024 Feb 28;74(1):94–113. doi: 10.1007/s00267-024-01943-z (PMC11208205; doi:10.1007/s00267-024-01943-z)
Supplement: Supplementary file 1 — Supplementary Materials [file 267_2024_1943_MOESM1_ESM.docx]

**Supplementary materials belonging to:**

**Participatory and Spatially Explicit Assessment to Envision the Future of Land-Use/Land-Cover Change Scenarios on Selected Ecosystem Services in Southwestern Ghana**

Evelyn Asante-Yeboah^a,b*^, HongMi Koo^a,c^, Mirjam A.F. Ros-Tonen^d^, Stefan Sieber^,b,e^, Christine Fürst^a,c^

^a^ Martin-Luther-University Halle-Wittenberg, Dept. Sustainable Landscape Halle (Saale), Germany. Email: [evelyn.asante-yeboah@student.uni-halle.de](mailto:evelyn.asante-yeboah@student.uni-halle.de), [hongmi.koo@geo.uni-halle.de](mailto:hongmi.koo@geo.uni-halle.de) [christine.fuerst@geo.uni-halle.de](mailto:christine.fuerst@geo.uni-halle.de)

^b^ Leibniz Centre for Agricultural Landscape Research, (ZALF), Eberswalder Str. 84, 15374 Müncheberg, Germany. Email: [stefan.sieber@zalf.de](mailto:stefan.sieber@zalf.de)

^c^ German Centre for Integrative Biodiversity Research (iDiv) Halle-Jena-Leipzig, Leipzig, Germany.

^d^ University of Amsterdam, Department of Geography, Planning and International Development Studies and Centre for Sustainable Development Studies, Amsterdam, The Netherlands, Email: [m.a.f.ros-tonen@uva.nl](mailto:m.a.f.ros-tonen@uva.nl)

^e^ Department of Agricultural Economics, Humboldt University of Berlin, 10099 Berlin, Germany.

*Corresponding author: [evelyn.asante-yeboah@student.uni-halle.de](mailto:evelyn.asante-yeboah@student.uni-halle.de), [eveyeb84@gmail.com](mailto:eveyeb84@gmail.com)

# Supplementary material 1: Protocol for the participatory scenario workshops

Two workshops were held, and both are captured in this section. The first workshop was held on March 16^th^, 2021, at the conference room of the Ahanta West Municipal Assembly, Agona Nkwanta. We started the workshop by explaining the results from the land-use/land-cover mapping exercise, for which we used geoinformation techniques and discussions with the local inhabitants (**Step 1** in Fig. 3 in the paper). We first discussed the structural dynamics (composition and spatial configuration) and land-cover transfers within the study landscape with the workshop participants using the land-use/land-cover maps generated in a previous study (Asante-Yeboah et al. 2022). We discussed the land-use/land-cover types captured in the map and the degree of land-use/land-cover transfers. We deliberated on the drivers of rapid land-use/land-cover changes and the impacts of its advancement (both in terms of potential and adverse effects).

In **Step 2**, three activities were done during the workshop. i) identify the ecosystem services relevant to the study landscape and indicator values, ii) establish the business-as-usual (BAU) scenario, and iii) develop the transitional probability rule set for the spatial simulations.

In **Activity 1**, A preliminary list of environmental services (ESs) was shared with the participants. We explained the ES concept to the participants in simple language as the benefit they derived from the ecosystem to meet their survival and livelihood needs and contribute to their well-being. To assist the participants in collectively selecting the locally relevant ESs, we used a Likert scale of 1 to 5 to assess the relevance of each identified ES to the needs of the participants (supplementary material 4). The ESs captured reflected the needs of the various actor groups and the contribution to their well-being. We also allowed the participants to express the capacity of each land-use/land-cover type to provide the identified ESs. We did this exercise using a Likert scale (supplementary material 5). We again discussed the values for the indicators using percentages. So, the participants were asked to collectively indicate what benefits (ESs) they derive from the landscape (captured as the indicator value and expressed in percentages of land cover types to provide ES). For the ESs that were not easy to assess based on perceptions, we used other methods to assess them outside the workshop window (for details, see the main paper).

In **Activity 2**, we discussed the two main drivers of rapid land-use/land-cover change (rubber and settlement expansion) revealed by the land-use/land-cover maps and change detention and intensity analyses. The participants agreed that these two drivers of land-use/land-cover change are strong and potentially strongly affect the provision of ESs.

In **Activity 3**, we developed the transitional rule set for simulating the BAU scenario. The rule sets were developed based on research participants’ perceptions of the likelihood of a land-use/land-cover type changing to either settlement or rubber plantation and the location of change. We used a Likert scale to facilitate the choice of the transitional rule set.

The second workshop (**Step 4** in the main paper) was held in May 2021. The workshop brought the research participants together to discuss the outcome of the simulations. We highlighted the risk of declining ES provisioning and the degradation of ESs as revealed by the simulations under the BAU scenarios. The workshop participants shared their life experiences with the current impacts of land-use/land-cover changes, especially those due to rubber and settlement expansion. All narratives were captured in the quotes cited in the main paper. The discussions during the second workshop necessitated the research participants to devise measures to address the landscape dynamics, such as the need for alternatives to detrimental land uses and land-use planning.

# Supplementary material 2: Description of land-use actors who participated in the two workshops (N=21)

| Land-use actors  N=21 | Level of engagement | Spatial level | Description |
| --- | --- | --- | --- |
| Farmers (n=6) and landowners (n=2) | Interest, influence, knowledge-based | Local | This category self-identified as (i) farmers cultivating food crops, rubber, and oil palm. (ii) land owners as the custodians of the land, who can be chiefs, clan heads, household heads, and/or farmers. The land-use decision depends on the landowner. |
| Department of Food and Agriculture (DoFA) (district level) (n=2) | Interest, knowledge-based | Local, district, national | DoFA is the body that oversees all agricultural activities. It provides technical support and introduces new technologies to farmers. It also coordinates with other stakeholders with an interest in the productivity of food production land uses (e.g, environmental NGOs). |
| Ghana Rubber Estate Limited (GREL) (n=2) | Interest, influence, knowledge-based | Local,  district, national | GREL is the company that enters into contractual agreements with farmers on rubber outgrower schemes. |
| Norpalm Ghana (n=2) | Interest, influence, knowledge-based | Local, district | This company deals with farmers cultivating oil palm and outgrower schemes. It supplies oil palm seedlings and buys oil palm fruits from farmers. |
| Land-Use and Spatial Planning Authority (Physical Planning Department) (n=2) | Interest, knowledge-based | District | This body provides technical advice and input to the Metropolitan, Municipal, and District Assemblies (MMDAs) in Ghana in preparing, demarcating, and approving land-use plans. |
| Forestry Commission; (n=1) Environmental Protection Agency (EPA) (n=1) | Interest, knowledge-based | District | These bodies provide technical advice in the preparation of land-use plans and are mandated to oversee the land uses in the district. |
| Scientific community, (n=1) Environmental NGO (n=1) | Interest, knowledge-based | District | These bodies facilitate knowledge transfers and novel ideas among various land users. |
| Real estate developer  n=1 | Interest, knowledge-based | District | Involved in acquiring land for commercial and infrastructural purposes. |

**Supplementary material 3: The preliminary list of ecosystem services selected for the studies**

| Ecosystem services | Description | Indicator/proxy | Data source | References |
| --- | --- | --- | --- | --- |
| Food provision | Wild and cultivated plants and terrestrial and aquatic animals for human nutrition | The proportion of products used as food for human consumption (%) | Stakeholder survey | (Haines-Young and Potschin-Young 2018) |
| Feed/forage provision | Use of wild and cultivated plants for animal nutrition | The proportion of products used as food for animal consumption (%) | Stakeholder survey | (Haines-Young and Potschin-Young 2018; Koo et al. 2019) |
| Marketable products | Products to generate household income | The proportion of products from a land-use/land-cover type sold for income (%) | Stakeholder survey | (Haines-Young and Potschin-Young 2018; Koo et al. 2019) |
| Fuelwood provision | Products for household energy/cooking | The proportion of products from a land-use/land-cover type used as fuelwood (%) | Stakeholder survey | (Haines-Young and Potschin-Young 2018; Schmidt et al. 2009; Koo et al. 2019) |
| Soil quality regulation | Litter production and decomposition process and effect on soil quality | The litter decomposition rate of a land-use/land-cover type | Decomposition rate values for species following Bakker et al., 2011, Wayarat 2015, N’Dri et al., 2018 | (Haines-Young and Potschin-Young 2018; N’Dri et al. 2018; Giweta 2020; Saj et al. 2021) |
| Species diversity | The diversity of species and varieties enabling the provision of ecosystem services | Type of species and amount of varieties there exist | Field data collection, stakeholder survey, literature | (MEA 2005; Haines-Young and Potschin-Young 2018; Omayio and Mzungu 2019) |
| Carbon sequestration | The ability of a land-use/land-cover type to sequester carbon | Amount of carbon sequestered per land-use/land-cover type using carbon lookup tables for Ghana (tCO_2_e) | Carbon lookup tables  Estimates from other research (rubber and palm) | (Haines-Young and Potschin-Young 2018, PASCO Corporation 2013) |
| Educational purposes | Characteristics of land-use/land-cover types that enable education and traditional knowledge transfer | Description of land-use/land-cover type and scoring based on its composition | Stakeholder survey | Haines-Young and Potschin 2018 |

# Supplementary material 4: Identification of locally relevant ecosystem services in southwestern Ghana (Ahanta West Municipal Assembly)

Kindly indicate the local relevance of each ecosystem service on a Likert scale (0-5)

| Ecosystem service | Description | Indicator/proxy | Low relevance | | Moderate relevance | | | High relevance | | |  |
| --- | --- | --- | --- | --- | --- | --- | --- | --- | --- | --- | --- |
|  |  |  | **1** | | **2** | | **3** | **4** | | **5** |  |
| Food provision | Wild and cultivated plants and terrestrial and aquatic animals for human nutrition | The proportion of products used as food for human consumption (%) |  |  | |  | | |  | | |
| Feed/forage provision | Use of wild and cultivated plants for animal nutrition | The proportion of products used as food for animal consumption (%) |  |  | |  | | |  | | |
| Marketable products | Products to generate household income | The proportion of products from a land-use/land-cover type sold for income (%) |  |  | |  | | |  | | |
| Fuelwood provision | Products for household energy/cooking | The proportion of products from a land-use/land-cover type used as fuelwood (%) |  |  | |  | | |  | | |
| Soil quality regulation | Litter production and decomposition process and effect on soil quality | The litter decomposition rate of a land-use/land-cover type |  |  | |  | | |  | | |
| Species diversity | The diversity of species and varieties enabling the provision of ecosystem services | Type of species and amount of varieties there exist |  |  | |  | | |  | | |
| Carbon sequestration | The ability of a land-use/land-cover type to sequester carbon | Amount of carbon sequestered per land-use/land-cover type using carbon lookup tables for Ghana (tCO_2_e) |  |  | |  | | |  | | |

# Supplementary material 5: Assessing the capacity of land-use/land-cover types to provide ecosystem services)

Kindly indicate the capacity to provide ecosystem services on a Likert scale of 1-5.

1 (No capacity), 2 ( Low capacity), 3 (Medium capacity), 4 (Moderate capacity), or 5 (High capacity) to provide the selected ecosystem services

| Ecosystem  services    Land-use types | Food provision | Marketable products | Fuelwood | Regulation of soil quality | Species diversity |
| --- | --- | --- | --- | --- | --- |
| Settlement |  |  |  |  |  |
| Rubber plantations |  |  |  |  |  |
| Palm vegetation |  |  |  |  |  |
| Cropland |  |  |  |  |  |
| Forest |  |  |  |  |  |
| Shrubland |  |  |  |  |  |
| Waterbody |  |  |  |  |  |
| Wetlands |  |  |  |  |  |

# Supplementary material 6: The steps in the applied Delphi method

1. We repeated steps 3 and 4 in a third round of scoring and finalized the values for each land-cover type.
2. We shared the summarized data from step 3 with the experts again, asked them to give a second opinion and evaluate their own response compared to the range provided, and gave them the chance to adjust their scores where necessary.
3. We reviewed the first round of scoring obtained in step 2. We summarized and calculated the mean and percentage range for each land-cover type.
4. We consolidated information from the research study and described the study area. We added land-cover maps and an explanation of each land-cover type and sent them out the experts for review.
5. We asked the experts to individually score the capacity of each land-cover type to contribute to soils quality regulation, by reflecting on litter production and decomposition processes on a percentage scale from 0 to 100.

# Supplementary material 7: Future scenario land-use/land-cover change pattern; the transition probability rule set

Please indicate in the table below the transition probability of land-use types changing to rubber or settlement and the possible influencing conditions of land-use change for the years 10, 30, and 50 years into the future.

Please use the Likert scale below

75-100%: Extremely probable

51-74%: Very probable

31-50%: Probable

11-30%: Not so probable

0-10%: Not probable

|  | **Current land-use/land-cover type** | **Target future land-use/land-cover types (settlement and rubber plantation)** | **Transition probability**  **iteration 2 (10 yrs)** | **Transition probability iteration 5 (25 yrs)** | **Transition probability, iteration 10 (50 yrs)** | **Influencing conditions** | |
| --- | --- | --- | --- | --- | --- | --- | --- |
|  |  |  |  |  |  | ***Neigh-boring land-use/land-cover type** | ****Attribute**  **conditions** |
| 1 | Shrubland | Rubber plantation |  |  |  |  |  |
|  |  | Settlement |  |  |  |  |  |
| 2 | Palm vegetation | Rubber plantation |  |  |  |  |  |
|  |  | Settlement |  |  |  |  |  |
| 3 | Cropland | Rubber plantation |  |  |  |  |  |
|  |  | Settlement |  |  |  |  |  |
| 4 | Forest | Rubber plantation |  |  |  |  |  |
|  |  | Settlement |  |  |  |  |  |
| 5 | Wetland | Rubber plantation |  |  |  |  |  |
|  |  | Settlement |  |  |  |  |  |
| 6 | Waterbody | Rubber plantation |  |  |  |  |  |
|  |  | Settlement |  |  |  |  |  |

* What neighboring land-use type will influence the highest probability of a land-use change?

** Which attributes or characteristics of the soil, environmental conditions, or surrounding areas will influence the highest probability of land-use/land-cover change? E.g., population, roads, towns, elevation, temperature, soil type.

**Attribute table**

|  | Current land-use/land-cover type | Target future land-use/land-cover type | Attribute conditions | | | Other attributes | |
| --- | --- | --- | --- | --- | --- | --- | --- |
|  |  |  | Slope  1: steep,  2: gentle,  3: flat | Soil type  (1: well-drained,  2: poorly drained,  3: rocky soil | Road type/road network  (1: good/tarred road, 2: good untarred road, 3: bad road network |  |  |
| 1 | Palm vegetation | Settlement |  |  |  |  |  |
|  |  | Rubber plantation |  |  |  |  |  |
| 2 | Cropland | Settlement |  |  |  |  |  |
|  |  | Rubber plantation |  |  |  |  |  |
| 3 | Forest | Settlement |  |  |  |  |  |
|  |  | Rubber plantation |  |  |  |  |  |
| 4 | Shrubland | Settlement |  |  |  |  |  |
|  |  | Rubber plantation |  |  |  |  |  |
| 5 | Waterbody | Settlement |  |  |  |  |  |
|  |  | Rubber plantation |  |  |  |  |  |
| 6 | Wetland | Settlement |  |  |  |  |  |
|  |  | Rubber plantation |  |  |  |  |  |
| 7 | Settlement | Other land use (indicate) |  |  |  |  |  |
|  | Rubber plantation | Other land use (indicate) |  |  |  |  |  |

**References**

Asante-Yeboah E, Ashiagbor G, Asubonteng K, et al. (2022) Analyzing variations in size and intensities in land use dynamics for sustainable land use management: A Case of the coastal landscapes of South-Western Ghana. Land 11:815

Giweta M (2020) Role of litter production and its decomposition, and factors affecting the processes in a tropical forest ecosystem: a review. j ecology environ 44:11. https://doi.org/10.1186/s41610-020-0151-2

Haines-Young R, Potschin-Young M (2018) Revision of the common international classification for ecosystem services (CICES V5. 1): a policy brief. One Ecosystem 3:e27108

Koo H, Kleemann J, Fürst C (2019) Impact assessment of land use changes using local knowledge for the provision of ecosystem services in northern Ghana, West Africa. Ecological Indicators 103:156–172

Millennium Ecosystem Assessment, 2005. Ecosystems and Human Well-being: Synthesis. Island Press, Washington, DC. Copyright © 2005 World Resources Institute. 155 pages

N’Dri JK, Guéi AM, Edoukou EF, et al. (2018) Can litter production and litter decomposition improve soil properties in the rubber plantations of different ages in Côte d’Ivoire? Nutr Cycl Agroecosyst 111:203–215. https://doi.org/10.1007/s10705-018-9923-9

PASCO Corporation, Japan. 2013. Forest Preservation Programme Report on Mapping of Forest Cover and Carbon Stock in Ghana. In collaboration with FC-RMSC, CSIR-FORIG and CSIR-SRI, Ghana. Saj S, Nijmeijer A, Nieboukaho J-DE, et al. (2021) Litterfall seasonal dynamics and leaf-litter turnover in cocoa agroforests established on past forest lands or savannah. Agroforest Syst 95:583–597. https://doi.org/10.1007/s10457-021-00602-0
